# The prevalence of methicillin-resistant *Staphylococcus aureus* among diabetic patients: a meta-analysis

**DOI:** 10.1007/s00592-019-01301-0

**Published:** 2019-04-06

**Authors:** Helen J. Stacey, Caitlin S. Clements, Susan C. Welburn, Joshua D. Jones

**Affiliations:** 1 Edinburgh Medical School, University of Edinburgh, Chancellor’s Building, 49 Little France Crescent, EH16 4SB Edinburgh, UK; 2 Division of Infection and Pathway Medicine, Edinburgh Medical School, Biomedical Sciences, University of Edinburgh, Chancellor’s Building, 49 Little France Crescent, EH16 4SB Edinburgh, UK; 3 International Campus, ZJU-UoE Institute, Zhejiang University School of Medicine, Zhejiang University, 718 East Haizhou Road, 314400 Haining, Zhejiang People’s Republic of China

**Keywords:** Diabetes, Diabetic patients, Meta-analysis, Methicillin-resistant *Staphylococcus aureus*, MRSA, Prevalence, Resistance

## Abstract

**Aims:**

Diabetic patients have multiple risk factors for colonisation with methicillin-resistant *Staphylococcus aureus* (MRSA), a nosocomial pathogen associated with significant morbidity and mortality. This meta-analysis was conducted to estimate the prevalence of MRSA among diabetic patients.

**Methods:**

The MEDLINE, Embase, BIOSIS, and Web of Science databases were searched for studies published up to May 2018 that reported primary data on the prevalence of MRSA in 10 or more diabetic patients. Two authors independently assessed study eligibility and extracted the data. The main outcomes were the pooled prevalence rates of MRSA colonisation and infection among diabetic populations.

**Results:**

Eligible data sets were divided into three groups containing data about the prevalence of MRSA colonisation or in diabetic foot or other infections. From 23 data sets, the prevalence of MRSA colonisation among 11577 diabetics was 9.20% (95% CI, 6.26–12.63%). Comparison of data from 14 studies that examined diabetic and non-diabetic patients found that diabetics had a 4.75% greater colonisation rate (*P* < 0.0001). From 41 data sets, the prevalence of MRSA in 10994 diabetic foot infection patients was 16.78% (95% CI, 13.21–20.68%). Among 2147 non-foot skin and soft-tissue infections, the MRSA prevalence rate was 18.03% (95% CI, 6.64–33.41).

**Conclusions:**

The prevalence of MRSA colonisation among diabetic patients is often higher than among non-diabetics; this may make targeted screening attractive. In the UK, many diabetic patients may already be covered by the current screening policies. The prevalence and impact of MRSA among diabetic healthcare workers requires further research. The high prevalence of MRSA among diabetic foot infections may have implications for antimicrobial resistance, and should encourage strategies aimed at infection prevention or alternative therapies.

**Electronic supplementary material:**

The online version of this article (10.1007/s00592-019-01301-0) contains supplementary material, which is available to authorized users.

## Introduction

*Staphylococcus aureus* infections can be classified as methicillin-resistant *Staphylococcus aureus* (MRSA) or methicillin-sensitive *Staphylococcus aureus* (MSSA). Individuals colonised with MRSA are typically asymptomatic; however, if the bacterium breaches the patient’s physical defences, infection can occur. The economic and clinical burden of MRSA is significant, with high rates of morbidity and mortality and increased hospital costs associated with MRSA compared to MSSA [1, 2]. Risk factors which increase susceptibility to MRSA colonisation include recent exposure to antimicrobial agents, sustained hyperglycaemia associated with diabetes mellitus (diabetes), hospitalisation within the past year, skin or soft-tissue infection (SSTI) on admission, and human immunodeficiency virus type one (HIV) infection [3]. Independently, MRSA colonisation has been associated with an increased risk of MRSA infection [4]. Certain patient groups, therefore, have multiple risk factors and a significantly increased susceptibility to MRSA colonisation and infection. These include, but are not limited to, patients with diabetes mellitus (diabetics), HIV-positive individuals, and haemodialysis patients [5, 6].

Diabetes is the most common metabolic disease in the world, and the global number of diabetics is predicted to rise from 382 million in 2013 to 592 million in 2035 [7]. Diabetes may be complicated by foot disease. In Scotland, 4.7% of diabetics are recorded as having had a foot ulcer and 0.7% as having had a lower limb amputation [8]. It has been estimated that, in England, approximately £1 of every £140 spent by the NHS goes towards the cost of caring for ulceration or amputation, and that the cost of treating diabetic foot is greater than that for any of the four most common cancers in the UK [9]. Gram-positive organisms are commonly identified from diabetic foot infections (DFIs), with *S. aureus* among those most commonly isolated. A 2010 systematic review of the prevalence of MRSA in diabetic foot infections estimated the prevalence to be 15–30% [10].

The rising prevalence of diabetes, predominantly in low- and middle-income countries, is occurring against the backdrop of globally increasing rates of antimicrobial resistance. Knowledge of the prevalence of colonisation or infection of diabetic patients by resistant pathogens, including MRSA, will therefore be important in assessing the extent to which interventions targeted towards diabetic patients may mitigate the spread of resistant pathogens.

Several meta-analyses have examined the prevalence of MRSA in high-risk populations, such as haemodialysis patients, HIV-positive patients and general, neonatal or paediatric intensive-care patients [5, 6, 11, 12]. However, to date, no meta-analysis has examined the prevalence of MRSA in diabetic patients. Therefore, the aim of this meta-analysis was to use studies of any design which reported data for 10 or more patients to estimate the prevalence of MRSA in diabetic patients.

## Methods

### Search strategy

Four electronic databases were searched for articles published up to 16 May 2018: EMBASE (1980–2018), Ovid MEDLINE^®^ Epub Ahead of Print, In-Process & Other Non-Indexed Citations, Ovid MEDLINE^®^ Daily, Ovid MEDLINE and Versions^®^ (1946–2018), Web of Science and the BIOSIS Citation Index (1926–2018). The Web of Science Core Collection Citation Indexes searched were: Science Citation Index Expanded (1900–2018), Conference Proceedings Citation Index- Science (1990–2018), Book Citation Index– Science (2005–2018) and the Emerging Sources Citation Index (2015–2018). The search was performed using the following terms: (“me??icillin-resistant *Staphylococcus aureus*” OR “MRSA” OR “?A-MRSA”) AND (“diabetes” OR “DFI” OR “diabetic foot” OR “diabetes mellitus type” OR “non?insulin dependent diabetes” OR “insulin?dependent diabetes” OR “?IDDM”) AND (“prevalence” OR “incidence” OR “epidemiology” OR “frequency” OR “occurrence” OR “rate” OR “predict*”). In Ovid, these terms were followed by the suffix ‘.mp.’ and they were searched as topics in Web of Science. Further articles were obtained using reference lists from a review article [10] and manual searching [13, 14]. A study protocol was not published prior to this study.

### Study selection criteria

All studies underwent title and abstract screening, eligible studies met the following criteria: (1) the patients had been diagnosed with diabetes mellitus and were colonised or infected with MRSA; (2) the study reported primary patient data on the prevalence of MRSA in 10 or more patients; (3) the study was published in the English language. There were no limitations on study date or type. Eligible studies were accessed in full-text to ensure that they fulfilled the inclusion criteria and provided sufficient data for the meta-analysis. Studies which could not be accessed in full-text, including presentation abstracts, were excluded; their authors were not contacted. MRSA prevalence data can be reported in terms of positive isolates or ulcers (potentially multiple per patient) and/or patients. For clarity, only studies which reported the prevalence of MRSA in terms of patient numbers were included. Title and abstract and full-text screening were performed independently by two authors (HJS and JDJ), with discrepancies resolved by consensus. Deduplication was performed using Endnote (version X8.0.1) and Zotero (version 5.0.47). This review was conducted in accordance with the PRISMA (Preferred Reporting Items for Systematic Reviews and Meta-Analyses) guidelines [15], and a PRISMA checklist completed (see additional file one).

### Data extraction and critical appraisal

The following information was extracted from each study: author(s), year of publication, country, study design, study setting, study population size, and number of MRSA-positive patients, whether the study reported on MRSA colonisation or infection and the type of infection.

All eligible studies were critically assessed using a modified Joanna Briggs Institute checklist for prevalence studies [16]. Publication bias was assessed using funnel plots. Funnel plots of sub-group analyses were not used to assess for publication bias, as funnel plot reliability decreases with fewer studies, particularly below ten [17].

### Statistical analysis

Random-effects meta-analyses were used throughout to calculate the pooled prevalence of MRSA in a given population with 95% confidence intervals (95% CIs). Study heterogeneity was assessed by the *I*^2^ statistic, reported with 95% CIs, and interpreted as low (≤ 25%), moderate (25–75%), or high (≥ 75%) [18]. All meta-analyses were carried out using MedCalc statistical software, version 18.0 (MedCalc Software, Ostend, Belgium). The significance of proportions was compared using the MedCalc N-1 Chi-squared calculator [19].

## Results

**Fig. 1:**
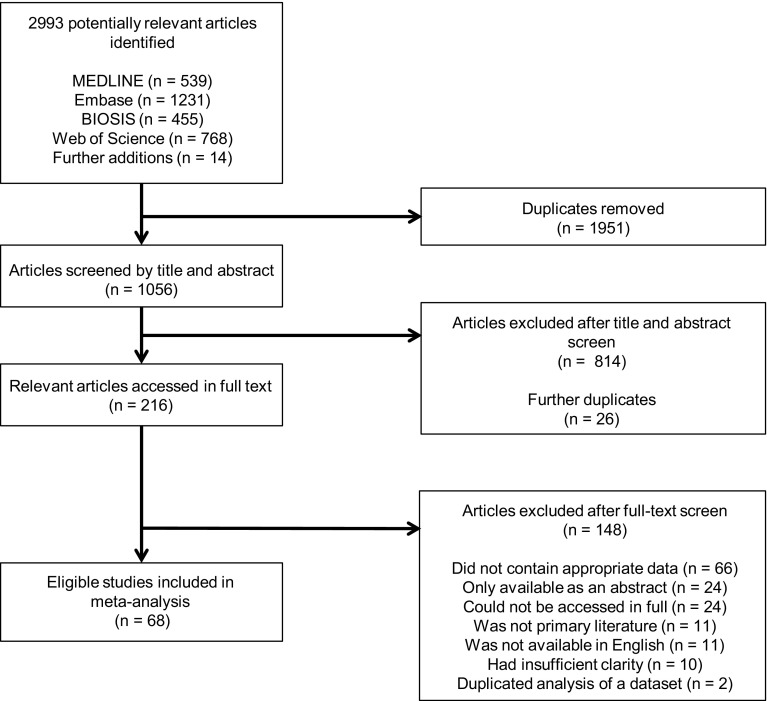
Flow diagram of study selection

After deduplication, systematic searching yielded 1056 articles published between March 1985 and May 2018. An additional 14 manuscripts were identified from a review article (*n* = 12) and manual searching [10, 13, 14]. Title and abstract screening identified 216 eligible articles, 148 of which were subsequently excluded after full-text screening. Articles were excluded, because they did not contain appropriate data (*n* = 66), were only available as an abstract (e.g. poster, presentation; *n* = 24), could not be accessed in full (*n* = 24), were not primary literature (*n* = 11), were not available in English (*n* = 11), had insufficient clarity for data extraction (*n* = 10), or duplicated analysis of another data set (*n* = 2). The study selection process is shown in Fig. 1.

A total of 68 studies were eligible for inclusion. Six studies contained two prevalence data sets; four contained data regarding the prevalence of MRSA in the nares and ulcers of diabetic foot infection (DFI) patients, another contained MRSA prevalence data for DFI and other SSTI patients, and another data regarding nasal colonisation or unspecified infection with MRSA among diabetic patients. There was, therefore, a total of 74 eligible data sets (Table 1). The data sets were categorised for subsequent meta-analysis: those with data about the prevalence of MRSA among DFIs (41/74), necrotizing fasciitis (NF) and other skin and soft-tissue infections (7/74), infections of unspecified source (3/74), and those with colonisation prevalence data (23/74). While critical appraisal of eligible studies highlighted shortcomings in reporting, it did not reveal further grounds to exclude any studies (see additional file two).

**Table 1:** Eligible data sets

Study	Study design	Study date(s)	Country	Setting	Mean age	% Male	Study population	MRSA +	%	95% CIs	Col/Inf
Goldstein et al. 1996 [55]	P cohort	1993–1994	USA	Ipat	NR	68	25	5	20.0	6.83–40.70	DFI
Tentolouris et al. 1999 [57]	Retro	NR	UK	Opat	61.5	NR	75	12	16.0	8.55–26.28	DFI
El-Tahawy 2000 [51]	Retro	1997–1999	Saudi Arabia	Ipat	NR	57	111	9	8.12	3.78–14.83	DFI
Saxena et al. 2002 [32]	P cohort	1996–1999	Saudi Arabia	Ipat (HD)	47.5*	45.7*	58	12	20.69	11.17–33.35	Col (N)
Ge et al. 2002 [54]	RCT	NR	USA	Opat	NR	NR	812	37	4.56	3.23–6.23	DFI
Von Baum et al. 2002 [36]	P cohort	1999–2002	Germany	Com (NH)	81.2*	29.5*	328	5	1.52	0.50–3.52	Col (N)
Dang et al. 2003 [49]	Retro	2000–2001	UK	Opat	NR	NR	63	19	30.16	19.23–43.03	DFI
Hartemann-Heurtier et al. 2004 [56]	P cohort	NR	France	Ipat	65	75	180	29	16.11	11.06–22.31	DFI
Lipsky et al. 2005 [59]	P cohort	1999–2001	Int	Ipat	61.5	54	103	5	4.85	1.60–10.97	DFI
Shankar et al. 2005 [66]	P cohort	2003–2004	India	Ipat	63	59.7	77	8	10.39	4.59–19.45	DFI
Daeschlein et al. 2006 [22]	P cohort	2003	Germany	Com (NH)	77.6*	16.2*	175	0	0.00	0.00–2.09	Col (N)
Gadepalli et al. 2006 [52]	P cohort	NR	India	Ipat	53.9	85	80	14	17.50	9.91–27.62	DFI
Sharma et al. 2006 [67]	Retro	2004–2005	Nepal	Ipat	61	55.8	43	0	0.0	0.00–8.22	DFI
Citron et al. 2007 [47]	Retro	2001–2004	USA	Ipat	NR	NR	433	48	11.09	8.29–14.43	DFI
Stanaway et al. 2007 [41]	P cohort	NR	UK	Opat	61	61	65	12	18.46	9.92–30.03	DFI
Stanaway et al. 2007 continued								11	16.92	8.76–28.27	Col (N)
Gorwitz et al. 2008 [21]	P cohort	2003–2004	USA	Com	NR	49	1125	18	1.60	0.95–2.52	Col (N)
Nather et al. 2008 [62]	P cohort	2005–2006	Singapore	Ipat	60	50	122	7	5.74	2.34–11.47	DFI
Richard et al. 2008 [78]	P cohort	2003–2004	France	Ipat	68	69.7	188	37	19.68	14.25–26.09	DFI
Aragon-Sanchez et al. 2008 [44]	Retro	2002–2007	Spain	I, O	64.7	62.7	185	35	19.89	14.26–26.56	DFI
Maghsoudi et al. 2008 [84]	P cohort	2000–2006	Iran	Ipat	53.4	40.4	94	0	0.00	0.00–3.85	Inf (Burns)
Sotto et al. 2008 [76]	P cohort	2004–2007	France	Ipat	68	61	513	48	9.36	6.98–12.21	DFI
Garazi et al. 2009 [38]	Cross-sectional	NR	USA	Com (NH)	83.4*	26.7*	51	18	35.29	22.43–49.93	Col (N)
Galkowska et al. 2009 [53]	P cohort	2006–2007	Poland	I, O	58	75.75	50	13	26.00	14.63–40.35	DFI
Lipsky et al. 2010 [60]	Retro	2003–2007	USA	Ipat	60 (med)	64	2220	165	7.43	6.38–8.60	DFI
Lipsky et al. 2010 continued					61 (med)	54.6	810	78	9.63	7.69–11.87	nfSSTI
Raju et al. 2010 [83]	P cohort	2004	India	I, O	NR	NR	110	14	12.73	7.14–20.43	Inf (NS)
Wang et al. 2010 [72]	Retro	2004–2006	China	Ipat	60.8	57.6	118	21	17.80	11.37–25.91	DFI
Changchien et al. 2011 [79]	Retro	2004–2008	Taiwan	Ipat	57.9	64	155	35	22.58	16.26–29.98	NF
Lipsky et al. 2011 [81]	Retro	Mixed	Int	Ipat	NR	NR	868	349^$^	40.21	36.93–43.56	nfSSTI
Lu et al. 2011 [3]	P cohort	2009	Taiwan	ED	NR	53.4*	124	9	7.26	3.37–13.33	Col (N)
Schechter-Perkins et al. 2011 [33]	P cohort	2009–2010	USA	ED	41*	47*	51	6	11.77	4.44–23.87	Col (N, Ph, H, G, P, W, C)
Tascini et al. 2011 [69]	P cohort	2006–2008	Italy	Opat	NR	NR	1295	76	5.87	4.65–7.29	DFI
Kutlu et al. 2012 [29]	P cohort	2006–2009	Turkey	Opat	56.28	46.4	304	30	9.87	6.76–13.79	Col (N)
Mendes et al. 2012 [61]	P cohort	2010	Portugal	I, O	62.7	42.9	49	25	51.02	36.34–65.58	DFI
Acharya et al. 2013 [42]	Retro	2003–2008	UK	Opat	66.2	66.9	130	20	15.39	9.66–22.76	DFI
Djahmi et al. 2013 [50]	P cohort	2011–2012	Algeria	Ipat	64*	62.3*	128	73	57.03	47.99–65.74	DFI
Fowler and Ilyas 2013 [80]	Retro	2005–2010	USA	Ipat	NR	NR	82	32	39.02	28.44–50.43	nfSSTI
Gupta et al. 2013 [24]	Retro	2008–2010	USA	Ipat	NR	95.7*	5019	554*	11.04	10.18–11.94	Col (N)
Haleem et al. 2013 [25]	Cross-sectional	NR	USA	I, O	55	64	79	7	8.86	3.64–17.41	Col (N)
Haleem et al. 2013 continued								7	8.86	3.64–17.41	DFI
Parriott and Arah 2013 [82]	Retro	NR	USA	Ipat	NR	0	28,949	17	0.06	0.03–0.09	Inf (NS)
Torres and Sampathkumar 2013 [34]	Mixed	2008	USA	Ipat	Nr	NR	336	33	9.82	6.86–13.52	Col (N)
Ahmed et al. 2014 [43]	P cohort	2007–2010	Egypt	Ipat	44.4*	N/A	52	9	17.31	8.23–30.33	DFI
Hennessey et al. 2014 [85]	P cohort	2000–2011	USA	Ipat	55* (med)	50.5*	54	15	27.78	16.46–41.64	Inf (Abdo)
Lavery et al. 2014 [58]	Retro	NR	USA	Ipat	59.55	NR	57	17	29.83	18.43–43.40	DFI
Yeoh et al. 2014 [35]	Retro	2010–2011	Singapore	Ipat (HD)	59.1*	54.5	139	25	17.99	11.99–25.40	Col (N, A, G)
Cervantes-Garcia et al. 2015 [46]	P cohort	2012–2013	Mexico	I, O	52.5	55	100	34	34.00	24.82–44.15	DFI
Cheng et al. 2015 [14]	Retro	1997–2013	Taiwan	Ipat	58.8*	67*	84	6	7.14	2.67–14.90	NF
Commons et al. 2015 [48]	P cohort	2012–2013	Australia	Ipat	54.4	60	177	77	43.50	36.08–51.15	DFI
Daeschlein et al. 2015 [23]	Retro	2006–2010	Germany	Ipat	59*	NR	1138	30	2.64	1.79–3.74	Col (N)
Hart et al. 2015 [26]	P cohort	2008–2011	Australia	Com	65.1	53.1	660	8	1.21	0.53–2.37	Col (N, A)
Jayarani and Sundarji 2015 [27]	Retro	2014–2015	Sri Lanka	Ipat	NR	70	100	40	40.00	30.33–50.28	Col (N)
Kao et al. 2015 [37]	P cohort	2008–2010	Taiwan	Ipat	69.4*	62.7*	376	78	20.75	16.76–25.20	Inf (NS)
Kao et al. 2015 continued								66	17.55	13.84–21.78	Col (N)
Karadag-Oncel et al. 2015 [28]	P cohort	2005 and 2013	Turkey	Opat	12.1	61.5	235	2	0.85	0.10–3.04	Col (N)
Lipsky et al. 2015 [77]	Retro	2011–2013	Int	I, O	61.7	57.2	201	83	41.29	34.41–48.44	DFI
Saltoglu et al. 2015 [64]	Retro	2011–2013	Turkey	I, O	61 (med)	68	455	7	1.54	0.62–3.14	DFI
Bravo-Molina et al. 2016 [45]	P cohort	2009–2013	Spain	Ipat	66	80	131	20	15.27	9.58–22.59	DFI
Gleeson et al. 2016 [13]	P cohort	NR	Ireland	Ipat	69*	55*	45	10	22.22	11.21–37.09	Col (N, P, W, C)
Reveles et al. 2016 [63]	Retro	2010–2014	USA	Ipat	52 (med)	69	318	47	14.78	11.07–19.17	DFI
Shettigar et al. 2016 [68]	P cohort	2011–2012	India	Ipat	60.5	72.5	200	52	26.00	20.07–32.66	DFI
Ashong et al. 2017 [74]	Retro	2011–2015	USA	Ipat	65 (med)	NR	158	31	19.62	13.74–26.68	DFI
Dunyach-Remy et al. 2017 [40]	P cohort	2010–2012	France	Ipat	68.5	72.5	276	17	6.16	3.63–9.68	DFI
Dunyach-Remy et al. 2017 continued								18	6.52	3.91–10.11	Col (N)
Lin et al. 2017 [39]	Cross-sectional	2014–15	China	Com	66.13	30.43	529	22	4.16	2.62–6.23	Col (N)
Shah et al. 2017 [65]	Cross-sectional	2014–15	Pakistan	Ipat	42.2	51.4	140	33	23.57	16.82–31.48	DFI
Wu et al. 2017 [73]	P cohort	2009–14	China	Ipat	NR	NR	312	21	6.73	4.21–10.11	DFI
Legese et al. 2018 [20]	P cohort	2016	Ethiopia	HCW	31.78*	41.3*	10	3	30.00	6.67–65.25	Col (N)
Henig et al. 2018 [75]	Retro	2012–15	USA	Ipat	58.4	64.3	648	224	34.57	30.91–38.37	DFI
Lin et al. 2018 [39]	P cohort	2015	Taiwan	I, O	NR	NR	354	10	2.83	1.36–5.13	Col (N)
Lin et al. 2018 continued				Ipat	NR	NR	112	27	24.11	16.53–33.10	DFI
van Asten et al. 2018 [70]	Retro	2010–14	USA	Ipat	53.8	76.9	143	11	7.69	3.90-13.35	DFI
Viquez-Molina et al. 2018 [71]	P cohort	2014–16	Costa Rica	Ipat	NR	NR	379	35	9.24	6.52–10.81	DFI

*med* median value, *Abdo* abdominal, *CIs* 95% confidence intervals, *Col (N, A, G, W, C, Ph, H)* colonisation (nasal, axilla, groin, surface wound, catheter, pharynx, hand), *Col* colonisation. *Com (NH)* community (nursing home), *DFI* diabetic foot infection, I, O, mixed in- and out- patient population, *Inf* infection, *Int* international, *Ipat (HD)* inpatient (haemodialysis), *NF* necrotizing fasciitis,* nfSSTI* non-foot skin and soft tissue infection, *NR* not reported, *NS* not specified. *Opat* outpatient, *P cohort* prospective cohort, *RCT* randomized controlled trial, *Retro* retrospective analysis

*Age or gender data were not available for the diabetic portion of the study population and are instead representative of a larger portion of or the whole study population

^$^combined data from three clinical trials. CIs, 95% confidence intervals

### The prevalence of MRSA colonisation among diabetic patients

**Fig. 2:**
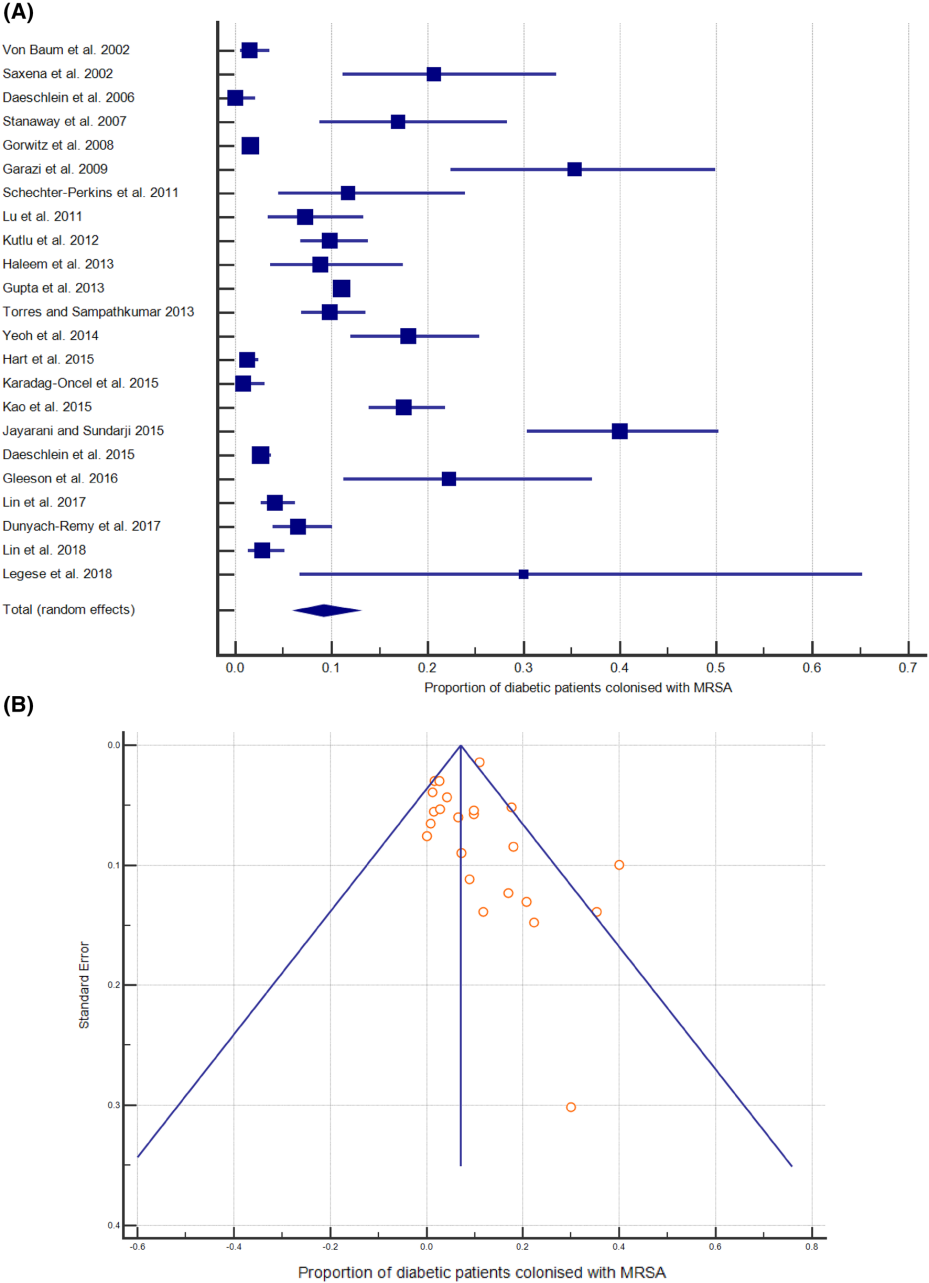
The prevalence of MRSA colonisation amongst diabetic patients. **a** Forest plot of the proportion of diabetic patients colonised with MRSA (*n* = 11577). **b** Funnel plot

Twenty-three data sets investigated the prevalence of MRSA colonisation among diabetic patients, published from 2002 to 2018 [13, 20–41]. The majority (19/23) examined MRSA carriage in the nares, one study examined the nares and axilla, another supplemented this with groin swabs, another with perineum and sites of wounds or catheters and another used multiple sites. Together, the 23 data sets represented a pooled population of 11577 diabetic patients with an MRSA colonisation rate of 9.20% (95% CI, 6.26–12.63%; Fig. 2a); heterogeneity among the studies was high (*I*^2^ = 96.38% [95% CI, 95.45–97.12%]). All forest plots are presented in the same way, the boxes show effect estimates for each study, weighted according to a random-effects model; the horizontal lines indicate 95% CIs; the centre of the diamond shows the pooled proportion and the horizontal tips represent 95% CIs. A funnel plot—on which the vertical line represents the summary estimate derived by meta-analysis and the diagonals represent 95% CIs around the summary effect—did not indicate publication bias (Fig. 2b). Inspection of the colonisation rates across the available 16 years did not reveal any correlation (*R*^2^ = 0.0052).

Sub-analyses of the data were conducted by patient setting and region or, where possible, nation (Table 2). Among the patient settings for which meta-analysis was possible, haemodialysis (HD) patients were found to have the highest colonisation rate (19.08%), followed by in-patients (13.46%), out- or emergency patients (8.33%), diabetics in nursing homes (6.61%), and diabetics in the community (2.19%). Only one study of 10 patients examined the prevalence of MRSA among diabetic healthcare workers (HCWs), of which 3/10 were MRSA-positive [20]. There were enough data to conduct regional and national analyses, including all patient settings, for East Asia, the Middle East, Germany, Taiwan, and the USA. The greatest colonisation prevalence was found in East Asia (12.85%) and the lowest in Germany (1.38%).

**Table 2:** Sub-group analyses of the prevalence of MRSA colonisation among diabetic patients

	Nation (s)	No. of data sets	No. of patients	Pooled prevalence of MRSA %	95% CI	*I*^2^ (%)	95% CI
Diabetic patients	–	23	11577	9.20	6.26–12.63	96.38	95.45–97.12
Healthcare workers	–	1	10	30^b^	N/A	N/A	N/A
Haemodialysis patients	–	2	197	19.08	13.93–24.81	0.00	0.00–0.00
In-patients^a^	–	7	7290	13.46	7.94–20.16	97.01	95.48–98.02
Out- or emergency patients	–	5	779	8.33	3.10–15.77	89.48	78.25–94.91
Nursing homes only	–	3	554	6.61	0.01–23.99	96.62	98.36
Community (excl. nursing homes)	–	3	2314	2.19	0.94–3.95	83.40	49.74–94.52
East Asia	China, Taiwan, Singapore, Sri Lanka	6	1622	12.85	5.34–23.00	96.40	94.21–97.77
Middle East	Saudi Arabia, Turkey	3	597	8.43	1.05–21.86	94.88	88.39–97.75
USA	–	6	6661	11.08	5.14–18.92	97.33	95.88–98.27
Taiwan	–	3	854	8.48	1.44–20.56	96.00	91.42–98.14
Germany	–	3	1641	1.38	0.27–3.33	80.23	37.66–93.73

^a^In-patients exclude haemodialysis patients, which were analysed separately

^b^Only one data set available

*CI* confidence interval

Many of the studies which contained colonisation data did so for diabetic patients as a sub-group of a wider cohort. Next, we, therefore, selected the 14 studies that contained colonisation data for diabetic and non-diabetic patients for comparison. Meta-analyses revealed that, among a pooled population of diabetic patients (*n* = 8975), there was an MRSA colonisation rate of 10.27% (95% CI, 6.27–15.12%; *I*^2^ = 96.80% [95.74–97.60%]). Among the comparative non-diabetic population (*n* = 38976), the colonisation rate was 5.52% (95% CI, 2.93–8.88%; *I*^2^ = 99.18% [99.01–99.32%]). The 4.75% difference between these colonisation rates was significant (*P* < 0.0001).

There were sufficient data among the 14 studies to perform sub-analyses for American (5/14), German (3/14), and East Asian (3/14) diabetics, as well as for nursing home residents (3/14) and in-patients (5/14). Across five studies, American diabetics (*n* = 6582) had a pooled MRSA carriage rate of 11.46% (95% CI, 4.84–20.42%; *I*^2^ = 97.87% [95% CI, 96.65–98.64%]) compared to 8.08% (95% CI, 2.82–15.72%; *I*^2^ = 99.66% [95% CI, 99.56–99.73%]) among 31005 non-diabetic Americans (*P* < 0.0001). Analysis of the three German studies revealed that 1641 diabetics had an MRSA carriage rate of 1.38% (95% CI, 0.27–3.33%; *I*^2^ = 80.23% [95% CI, 37.66–93.73%]), this was greater than the comparative rate of 0.84% (95% CI, 0.40–1.45%; *I*^2^ = 73.57% [95% CI, 11.49–92.11%]) for 5883 non-diabetics in these studies (*P* = 0.047). Comparison across the three East Asian studies revealed that 639 diabetics had a carriage rate of 14.34% (95% CI, 8.46–21.46%; *I*^2^ = 79.70% [95% CI, 35.60–93.60%]), compared to 7.65% (95% CI, 0.84–20.40%; *I*^2^ = 96.74% [95% CI, 93.33–98.41%]) in the comparative population of 1296 non-diabetics (*P* < 0.0001). Among the five studies that contained data on 6914 in-patient diabetics (excluding HD patients), there was a colonisation rate of 11.17% (95% CI, 5.78–18.04%; *I*^2^ = 97.18% [95% CI, 95.39–98.28%]), compared to 7.43% (95% CI, 3.02–13.59%; *I*^2^ = 99.08% [95% CI, 98.70-99.34%]) among 17072 non-diabetic in-patients (*P* < 0.0001). Three studies contained data regarding 554 nursing home residents, with a pooled MRSA prevalence rate of 6.61% (95% CI, 0.01–23.99%; *I*^2^ = 96.62% [95% CI, 93.01–98.36%]). This was similar to the 6.62% (95% CI, 0.23–20.71%; *I*^2^ = 97.93% [95% CI, 96.15–98.89%]) carriage rate among the comparative population of 3298 non-diabetic residents examined by these studies (*P* = 0.993).

### The prevalence of MRSA among patients with DFI

**Fig. 3:**
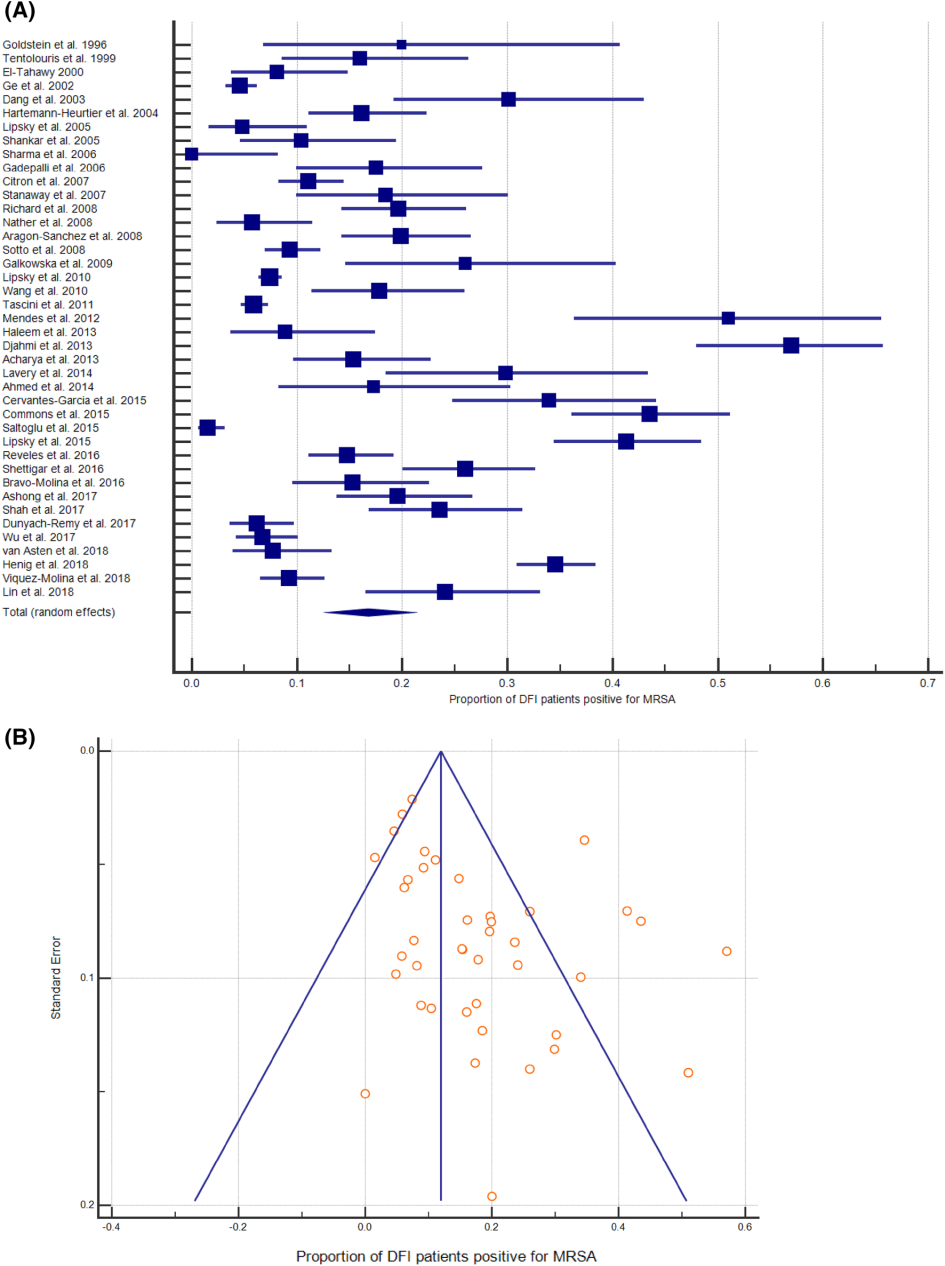
The prevalence of MRSA amongst DFI patients. **a** Forest plot of the proportion of MRSA-positive DFI patients (*n* = 10994). **b** Funnel plot

Data about the prevalence of MRSA among DFI patients were contained in 41 data sets [42–50,40,51–68,41,69–75,39,76,77,25,78], representing 10994 patients, with an MRSA prevalence rate of 16.78% (95% CI, 13.21–20.68%; Fig. 3a). Inter-study variability was high (*I*^2^ = 96.16% [95% CI, 95.43–96.77%]). A funnel plot of these studies was weakly asymmetrical (Fig. 3b). Analysis of the prevalence by publication year did not reveal any trend (*R*^2^ = 0.0487).

Sub-analyses by patient setting, region or nation were conducted (Table 3). These revealed that the 2.82% difference in the proportion of DFI out- and in-patients that were MRSA-positive was significant (*P* = 0.0008). There were sufficient data to perform sub-analyses of all patient settings for five broad regions, of which Central America had the highest (20.07%) and East Asia the lowest (12.73%) prevalence of MRSA. Among the five nations for which there were sufficient data to perform national sub-analyses, the UK had the highest prevalence (19.59%), while China had the lowest (11.65%).

**Table 3:** Sub-group analyses of the prevalence of MRSA amongst DFI patients

	Nation(s)	No. of data sets	No. of patients	Pooled prevalence %	95% CI	I^2^ (%)	95% CI
DFI patients	–	41	10994	16.78	13.21–20.68	96.16	95.43–96.77
Out-patients	–	6	2440	13.16	7.88–19.55	91.99	85.34–95.63
In-patients	–	28	7444	15.98	11.99–20.42	95.59	94.51–96.47
Central America	Costa Rica, Mexico	2	479	20.07	2.45–48.64	96.81	91.55–98.80
North Africa & The Middle East	Algeria, Egypt, Saudi Arabia, Turkey	4	746	17.25	0.63–49.16	98.62	97.83–99.12
Western Europe	France, Italy, Portugal, Spain	7	2295	16.96	9.77–25.63	94.96	91.83–96.89
Indian Subcontinent	India, Nepal, Pakistan	5	540	14.40	6.43–24.87	89.11	77.32–94.77
East Asia	China, Singapore, Taiwan	4	664	12.73	5.64–22.15	89.88	76.99–95.55
UK	–	4	333	19.59	13.88–26.01	49.56	0.00-83.31
India	–	3	357	18.34	10.06–28.44	78.91	32.51–93.41
USA	–	10	4893	14.70	8.54–22.18	97.29	96.25–98.05
France	–	4	1157	12.28	7.18–18.51	88.04	71.78–94.93
China	–	2	430	11.65	3.14–24.56	90.40	65.19–97.35

*CI* confidence interval

### The prevalence of MRSA among patients with NF or SSTIs and unspecified infections

Seven data sets examined the prevalence of MRSA in NF (*n* = 2), burns (*n* = 1), and non-foot SSTI (*n* = 4), and three further studies did not specify the type of infection in question [14, 37, 60, 79–85]. The NF and non-foot SSTI studies were grouped together for analysis and represented a population of 2147 patients with an MRSA prevalence rate of 18.03% (95% CI, 6.64–33.41%; *I*^2^ = 98.20% [95% CI, 97.44–98.73%]). The three studies that did not specify the nature of the MRSA infection studied represented 29435 patients with a prevalence rate of 8.08% (95% CI, 0.30–34.80%; *I*^2^ = 99.43% [95% CI, 99.14–99.62%]).

## Discussion

Diabetes mellitus is the most prevalent metabolic disease worldwide and has been reported to be a risk factor for MRSA colonisation at the time of admission to hospital [86]. The MRSA colonisation rates of diabetic in- and out-patients in this study were 13.46% and 8.33%, respectively. These are higher than many national estimations; for example, the nasal MRSA colonisation rate has been estimated to be 2.6% in Japan [87], 3.9% among healthy Chinese children [88] and up to 2.1% across nine European countries [89]. In Scotland, a colonisation rate of 3.8% on admission has been observed, rising to 20% among patients admitted to nephrology, care of the elderly, dermatology, and vascular surgery—specialties likely to see high numbers of diabetic patients [90]. The MRSA colonisation rate among diabetics in the community identified by this study was therefore generally comparable with values for broader populations (2.19%). National sub-analyses showed that, in America, diabetics had a carriage rate 7.4 times higher than the previously estimated national prevalence rate of 1.5% [21]. While, in Germany, diabetics had an MRSA colonisation rate 1.5 times higher than has recently been estimated for the wider German population [91], although more studies of MRSA carriage among German diabetics are needed to confirm this as only three were available.

The levels of MRSA colonisation among diabetics in nursing homes or the wider community, in-patients and out-patients suggest MRSA carriage reflects the extent of interaction with the healthcare system. It is difficult to compare the values obtained here for in- and out-patients to data regarding the prevalence of MRSA colonisation among general hospital populations as such studies offer a variety of results that typically reflect different patient populations and contexts [92–95]. However, this study compared the MRSA colonisation rates for diabetic and non-diabetic patients across the 14 studies that contained data for both. This showed that diabetics had a significantly higher colonisation rate than non-diabetics, a finding which held for sub-analysis of in-patient, American, German, or East Asian populations. Notably, the rates of MRSA carriage among diabetic and non-diabetic nursing home residents were very similar, supporting the above suggestion that MRSA carriage rates reflect the extent of interaction with a healthcare environment.

Further sub-analyses showed that diabetic HD patients had the greatest colonisation rate (19.08%; *n* = 197). The colonisation rate of non-diabetic HD patients has been previously estimated by meta-analysis to be 6.2% (*n* = 5596; [11]). The higher finding here likely reflects the synergy of two high-risk states and differences in study population size. However, the MRSA colonisation rate among diabetic patients in this study (9.20%) exceeded the previous meta-analytic estimates of the colonisation rate among the other high-risk groups, e.g. 6.9% among 6,558 HIV-1 patients and 7% and 1.9% among 63740 general and 19722 neonatal or paediatric intensive-care patients, respectively [6, 11, 12].

Only one study investigated the MRSA colonisation rate among diabetic HCWs, showing that it exceeded that of non-diabetic HCWs (30% [*n* = 10] vs. 5.8% [*n* = 242]; [20]. A previous meta-analysis of MRSA colonisation among HCW in Europe and the USA has estimated carriage at 1.8–4.4%, with nurses reported have a carriage rate of 6.9% with an odds ratio of 2.58 compared to other HCWs [96]. The increased carriage risk for nurses has been recorded elsewhere, and intensive patient contact is thought to be a risk factor [97, 98]. The significance of potentially greater rates of MRSA colonisation of diabetic HCWs requires further investigation, especially given the high prevalence of obesity among some sectors of the healthcare workforce [99].

Diabetic foot infections can be mono- or poly-microbial, and may be caused by a wide range of pathogens, including MRSA. This study found that the prevalence of MRSA among 10994 DFI patients was 16.78%. This is comparable to the prevalence estimated by a 2010 systematic review [10]. A higher prevalence was identified for in-patients compared to out-patients, likely reflecting the difference in extent of interaction with the healthcare system. From the UK, one eligible study reported MRSA colonisation data among diabetics at a value lower than the proportion of MRSA-positive UK DFI patients (16.9% vs. 19.59%). Further work is therefore needed in the UK to clarify the extent to which diabetic patients are colonised with MRSA.

This study was limited by the small number of eligible studies, such that only sub-analyses based on patient setting or geographic region could be conducted. Much of the often high level of heterogeneity observed in individual sub-analyses therefore likely reflects variation in other factors not simultaneously subject to sub-analysis. Sub-analyses by geographic region, although useful for appraising broad trends, are low resolution given the complexity of factors that underlie MRSA prevalence. While there were enough studies to conduct some national sub-analyses, many included low numbers of studies and were also subject to influence by local inter-study variation. More studies, in particular of the prevalence of MRSA colonisation among diabetic patients, are needed to provide a greater evidence base and permit finer stratification of data in future analyses. The variety of detection techniques used by eligible studies may have led to a slight underestimation, with PCR and chromogenic culture media being more sensitive than nonchromogenic media [100].

Meta-analyses are designed to be reproducible and identify as many eligible manuscripts as possible. However, this may be limited by the omission of some relevant manuscripts, because they do not contain the appropriate terms in the fields searched or were incorrectly indexed [101]. During this study, we became aware of two manuscripts that contained pertinent data, but were not detected by our systematic search as they lacked terms related to diabetes [13] or MRSA [14] in the searched fields. Given the topic of this study, it was not feasible to amend the search strategy to omit either terms related to diabetes or MRSA. Therefore, although we were unable to identify these two studies systematically or through review articles, we recognised that they contained relevant data and included them as ‘manual’ search results. This transparent approach enhances the reproducibility of this study, while acknowledging some of the inherent limitations of systematic approaches.

This study may also be limited by not being pre-registered. Registration of reviews is a non-essential recommendation designed to encourage transparency, improve quality and reduce duplication. Pre-registration of reviews that are never completed is not recommended [102]. This study was conceived as a student project, many of which are not published, and the authors therefore decided it was inappropriate to register retrospectively. However, the authors are not aware of any similar studies underway and complied with the PRISMA statement throughout.

Taken together, the results of this study suggest that diabetics interacting with the healthcare system are likely to have a higher rate of MRSA colonisation than non-diabetics, raising the question of whether diabetic staff and patients should be subject to targeted screening. Targeted screening of high-risk patients has been shown to offer a cost-effective, efficacious, alternative to universal screening [103–105]. In the UK, patients admitted to high-risk specialties or with previous MRSA colonisation or infection are targeted for screening [106]. Given the rates of colonisation and infection recorded in this analysis, many diabetic patients may, therefore, be eligible for screening under the current policy. However, research is warranted to evaluate the potential benefits of amending current guidelines to specifically and proactively target colonisation among high-risk patient groups, such as diabetics. While screening of staff is not current policy, more work is required to evaluate the potential benefits of screening diabetic HCWs in reducing the spread of MSRA. However, any screening of specific patient or staff groups must avoid stigmatisation. Finally, the high prevalence of MRSA among DFI patients and consequent long-term antibiotic administration has wider implications for antimicrobial resistance. While the prevention of such infections should remain the goal, with increasing levels of antimicrobial resistance, it is also important that alternative therapies are investigated [107].

## Electronic supplementary material

Below is the link to the electronic supplementary material.

Supplementary material 1 (DOCX 28 KB)

Supplementary material 2 (DOCX 36 KB)

## Data Availability

Data sharing is not applicable to this article as no data sets were generated or analysed during the current study.

## Abbreviations

CIs: 95% confidence intervals
HIV-1: Human immunodeficiency virus type one
MRSA: Methicillin-resistant *Staphylococcus aureus*
NF: Necrotizing fasciitis
PCR: Polymerase chain reaction
PRISMA: Preferred reporting items for systematic reviews and meta-analyses
SSTI: Skin and soft-tissue infection
