# Supplementary material for: The prevalence of methicillin-resistant Staphylococcus aureus among diabetic patients: a meta-analysis
Source: Acta Diabetol. 2019 Apr 6;56(8):907–21. doi: 10.1007/s00592-019-01301-0 (PMC6597605; doi:10.1007/s00592-019-01301-0)
Supplement: Supplementary file 2 — Supplementary material 2 (DOCX 36 KB) [file 592_2019_1301_MOESM2_ESM.docx]

**Critical appraisal**A modified Joanna Briggs Institute checklist for prevalence studies was used. The original checklist is available at <http://joannabriggs.org/assets/docs/critical-appraisal-tools/JBI_Critical_Appraisal-Checklist_for_Prevalence_Studies2017.pdf> [accessed 24.10.18].

| Study | Was the sample frame appropriate to address the target population? | Were study participants sampled in an appropriate way? | Was the sample size adequate for this analysis? (i.e. 10<) | Were the study subjects and the setting described in detail? | Was the data analysis conducted with sufficient coverage of the identified sample? | Were valid methods used for the identification of MRSA? | Was the presence of MRSA measured in a standard, reliable way for all participants? | Was there appropriate statistical analysis? | Was the response rate adequate, and if not, was the low response rate managed appropriately? | Comment(s) |
| --- | --- | --- | --- | --- | --- | --- | --- | --- | --- | --- |
| Acharya *et al.* 2013 | Y | Y | Y | Y | Y | Y* | Y | Y | N/A | *Swab methodology not detailed. |
| Ahmed *et al.* 2014 | Y | Y | Y | N* | Y | Y^ | Y | Y | N/A | *Detailed demographic data not reported. ^Swab methodology not described. |
| Aragon-Sanchez *et al.* 2008 | Y | Y | Y | Y | Y | Y | Y | Y | N/A |  |
| Ashong *et al.* 2017 | Y | Y | Y | Y | Y | Y | Y | Y | N/A |  |
| Bravo-Molina *et al.* 2016 | Y | Y | Y | Y | Y | Y | Y | Y | N/A |  |
| Cervantes-Garcia *et al.* 2015 | Y | Y | Y | Y | Y | Y | Y | Y | N/A |  |
| Changchien *et al.* 2011 | Y | Y | Y | Y | Y | Y | Y | Y | N/A |  |
| Cheng *et al.* 2015 | Y | Y | Y | N* | Y | Y^ | UC" | Y | N/A | *Demographic data for diabetic sub-population not reported. ^Swab methodology not described. "Unclear if microbiology conducted in the same way for all patients. |
| Citron *et al.* 2007 | Y | UC* | Y | N | Y | Y | Y | Y | N/A | *Study contains data from three previous clinical trials that individually contain sampling and demographic details. |
| Commons *et al.* 2015 | Y | Y | Y | Y | Y | Y | Y | Y | N/A |  |
| Daeschlein *et al.* 2006 | Y | UC | Y | N* | Y | Y | Y | Y | N/A | *Demographic data for diabetic sub-population not reported. |
| Daeschlein *et al.* 2015 | Y | Y | Y | N* | Y | Y | Y | Y | N/A | *Demographic data for diabetic sub-population and study population % male not reported. |
| Dang *et al.* 2003 | Y | Y | Y | N* | Y | Y^ | Y | Y | N/A | *Demographic data not reported. ^Swab methodology not described. |
| Djahmi *et al.* 2013 | Y | Y | Y | N* | Y | Y | Y | Y | N/A | *Demographic data for diabetic sub-population not reported. |
| Dunyach-Remy *et al.* 2017 | Y | Y | Y | Y | Y | Y | Y | Y | N/A |  |
| El-Tahawy 2000 | Y | Y | Y | Y | Y | Y | Y | Y | N/A |  |
| Exiara *et al.* 2009 | Y | Y | Y | Y | Y | Y | Y | Y | N/A |  |
| Fowler and Ilyas 2013 | Y | Y | Y | N* | Y | Y | UC^ | Y | N/A | *Demographic data not reported. ^Unclear if microbiology conducted in the same way for all patients. |
| Gadepalli *et al.* 2006 | Y | UC | Y | N* | Y | Y | Y | Y | N/A | *Study dates not reported. |
| Galkowska *et al.* 2009 | Y | UC | Y | Y | Y | Y | Y | Y | N/A |  |
| Garazi *et al.* 2009 | Y | Y | Y | N* | Y | Y | Y | Y | N/A | *Study dates not reported. Study dates and demographic data for diabetic sub-population not reported. |
| Ge *et al.* 2002 | Y | UC* | Y | N^ | Y | Y | Y | Y | N/A | *Patients enrolled on two clinical trials, selection not described. ^Study dates and demographic data not reported. |
| Gleeson *et al.* 2016 | Y | Y | Y | N* | Y | Y | Y | Y | N/A | *Study dates and demographic data for diabetic sub-population not reported. |
| Goldstein *et al.* 1996 | Y | Y | Y | N* | Y | Y | Y | Y | N/A | *Age data not reported. |
| Gorwitz *et al.* 2008 | Y | Y | Y | N* | Y | Y | Y | Y | N/A | *Age data for diabetic sub-population not reported. |
| Gupta *et al.* 2013 | Y | Y | Y | N* | Y | Y | Y | Y | N/A | *% male data for diabetic sub-population not reported; age data not clearly reported. |
| Haleem *et al.* 2013 | Y | Y | Y | N* | Y | Y | Y | Y | N/A | *Study dates not reported. |
| Hart *et al.* 2015 | Y | Y | Y | Y | Y | Y | Y | Y | N/A |  |
| Hartemann-Heurtier *et al.* 2004 | Y | Y | Y | N* | Y | Y | Y | Y | N/A | *Study dates not reported. |
| Henig *et al.* 2018 | Y | Y | Y | Y | Y | Y | Y | Y | N/A |  |
| Hennessey *et al.* 2014 | Y | Y | Y | N* | Y | Y^ | Y | Y | N/A | *Study dates and demographic data for diabetic sub-population not reported. ^Precise swab and culture methodology not described. |
| Jayarani and Sundarji 2015 | Y | Y | Y | N* | Y | Y | Y | Y | N/A | *Age data not clearly reported. |
| Jude *et al.* 1999 | Y | Y | Y | N* | Y | Y | Y | Y | N/A | *% male and study dates not reported. |
| Kao *et al.* 2015 | Y | Y | Y | N* | Y | Y | Y | Y | N/A | *Study dates and demographic data for diabetic sub-population not reported. |
| Karadag-Oncel *et al.* 2015 | Y | Y | Y | Y | Y | Y | Y | Y | N/A |  |
| Kutlu *et al.* 2012 | Y | Y | Y | Y | Y | Y | Y | Y | N/A |  |
| Lavery *et al.* 2014 | Y | Y | Y | N* | Y | Y | Y^ | Y | N/A | *Study dates not reported. ^Swab methodology not detailed. |
| Legese *et al.* 2018 | Y | UC | Y | N^ | Y | Y | Y | Y | N/A | *Demographic data for diabetic sub-population not reported. |
| Lin *et al.* 2017 | Y | Y | Y | Y | Y | Y | Y | Y | N/A |  |
| Lin *et al.* 2018 | Y | UC | Y | Y | Y | Y* | Y | Y | N/A | *Swab methodology not detailed. |
| Lipsky *et al.* 2005 | Y | Y | Y | N* | Y | Y | Y | Y | N/A | *Participating sites not detailed. |
| Lipsky *et al.* 2010 | Y | Y | Y | Y | Y | Y | Y | Y | N/A |  |
| Lipsky *et al.* 2011 | Y | Y* | Y | Y | Y | Y | Y | Y | N/A | *Participants pooled from three cited clinical trials. |
| Lipsky *et al.* 2015 | Y | Y | Y | Y | Y | Y | Y | Y | N/A |  |
| Lu *et al.* 2011 | Y | Y | Y | N* | Y | Y | Y | Y | N/A | *Demographic data for diabetic sub-population not reported. |
| Maghsoudi *et al.* 2008 | Y | Y | Y | Y | Y | UC* | Y | Y | N/A | *Microbiological procedures not specified. |
| Mendes *et al.* 2012 | Y | Y | Y | Y | Y | Y | Y | Y | N/A |  |
| Nather *et al.* 2008 | Y | Y | Y | Y | Y | UC* | UC | Y | N/A | *Microbiological procedures not specified. |
| Parriott and Arah 2013 | Y | Y | Y | N* | Y | Y | Y | Y | N/A | *Age data and study dates not reported. |
| Raju *et al.* 2010 | Y | Y | Y | N* | Y | Y | UC^ | Y | N/A | *Demographic data not reported. ^Nature of microbiological sample collection not specified. |
| Reveles *et al.* 2016 | Y | Y | Y | Y | Y | Y | Y | Y | N/A |  |
| Richard *et al.* 2008 | Y | Y | Y | Y | Y | Y | Y | Y | N/A |  |
| Saltoglu *et al.* 2015 | Y | Y | Y | Y | Y | Y | Y | Y | N/A |  |
| Saxena *et al.* 2002 | Y | Y | Y | N* | Y | Y | Y | Y | N/A | *Demographic data for diabetic sub-population not reported. |
| Schechter-Perkins *et al.* 2011 | Y | Y | Y | N* | Y | Y | Y | Y | N/A | *Demographic data for diabetic sub-population not reported. |
| Shah *et al.* 2017 | Y | Y | Y | Y | Y | Y* | Y | Y | N/A | *Swab methodology not detailed. |
| Shankar *et al.* 2005 | Y | UC | Y | Y | Y | Y | Y | Y | N/A |  |
| Shettigar *et al.* 2016 | Y | Y | Y | Y | Y | Y | Y | Y | N/A |  |
| Sotto *et al.* 2008 | Y | UC | Y | Y | Y | Y | Y | Y | N/A |  |
| Stanaway *et al.* 2007 | Y | Y | Y | N* | Y | Y | Y | Y | N/A | *Study dates not reported. |
| Tascini *et al.* 2011 | Y | Y | Y | N* | Y | Y | Y | Y | N/A | *Demographic data not reported. |
| Torres and Sampathkumar 2013 | Y | Y | Y | N* | Y | Y | Y | Y | N/A | *Demographic data not reported. |
| van Asten *et al.* 2018 | Y | Y | Y | Y | Y | UC* | Y | Y | N/A | *Microbiological procedures not specified. |
| Von Baum *et al.* 2002 | Y | Y | Y | N* | Y | Y | Y | Y | N/A | *Study dates and demographic data for diabetic sub-population not reported. |
| Viquez-Molina *et al.* 2018 | Y | Y | Y | N* | Y | Y | Y | Y | N/A | *Demographic data not reported. |
| Wang *et al.* 2010 | Y | Y | Y | Y | Y | Y | Y | Y | N/A |  |
| Wu *et al.* 2017 | Y | Y | Y | N* | Y | Y | Y | Y | N/A | *Demographic data not reported. |
| Yeoh *et al.* 2014 | Y | Y | Y | N* | Y | Y | Y | Y | N/A | *Demographic data for diabetic sub-population not reported. |
